# Supplementary material for: A Small Gtp-Binding Protein GhROP3 Interacts with GhGGB Protein and Negatively Regulates Drought Tolerance in Cotton (Gossypium hirsutum L.)
Source: Plants (Basel). 2022 Jun 15;11(12):1580. doi: 10.3390/plants11121580 (PMC9227279; doi:10.3390/plants11121580)
Supplement: Supplementary file 1 [file plants-11-01580-s001.zip › plants-1730298-supplementary.pdf]

Table S1. Primer and application.

Primer	Sequence（5′-3′）	Application
CM-GhROP3-F	TCTAGAGGATCCCAGAGCTAAGACATTA	Vector construction
CM-GhROP3-R	CCCGGGTTGCAAGGCTTTCTCTTTGG	
Y2H-GhROP3-F	CCCGGGATGAGCACTGCAAGATTTATC	
Y2H-GhROP3-R	CTGCAGTCAAAGGAAAGCACATGTTCT	
Y2H-GhGGB-F	GAATTCATGGCGACGAGAGAGTGGAAC	
Y2H-GhGGB-R	GGATCCCCTAAATTATACCTATGGCAGT	
BiFC-GhROP3-F	CTCGAGATGAGCACTGCAAGATTTATC	
BiFC-GhROP3-R	GCGGCCGCTCAAAGGAAAGCACATGTTCT	
BiFC-GhGGB-F	CTCGAGATGGCGACGAGAGAGTGGAAC	
BiFC-GhGGB-R	GCGGCCGCCAATTATACCTATGGCAGT	
q-GhROP3-F	GAGCAACACCAATATCAACATCT	qRT-PCR
q-GhROP3-R	GGCCTCAAAGCTACTTTTATTG	
GhUBQ7-F	GAAGGCATTCCACCTGACCAAC	
GhUBQ7-R	CTTGACCTTCTTCTTCTTGTGCTTG	
GhCLA1-F	GCCCTTTGTGCATCTTC	
GhCLA1-R	CTCTAGGGGCATTGAAG	
GhNCED2-F	GACTCGCCGTGCCATTATTT	
GhNCED2-R	CGACTTTGGCGAATCCTGAC	
GhNCED3-F	TGGGTTGAATCCCCTGATACTT	
GhNCED3-R	GCGACGTGTGGACTTACCTGT	
GhNCED8-F	TGCATGACACCACCAGATTCCA	
GhNCED8-R	TAATGGCACGGCGAGTCGATTT	
GhNCED9-F	CCCAGTAGACGGTCCGATAA	
GhNCED9-R	ATCCCAAACCTTGACATCTTG	
GhZEP-F	CTTCCCGCAAACAACATTCG	
GhZEP-R	GCCTTCCTCCTGCCATTATCA	
GhABI2-F	AGTCAAATCCTCAAAGCCGAAAGTG	
GhABI2-R	ACAATAGTTCGCAACCTGACACCCT	
GhABI5-F	GTGGGTCCGAGTTTTGTTCC	

GhABI5-R	AGCTGCACCTACTTTTCCGT
GhABF2-F	CCAATTTGCTGGGAAGGATAA
GhABF2-R	GGTAACTGGTGCTGGTTTGATT
GhABF4-F	CCAGTCTTTCCAGAGCAGCA
GhABF4-R	TCCCATCCCACCACTCTGAA
GhCYP707A-F	TCTTTGAATCCCCCTTACACTTGA
GhCYP707A-R	TGCCGCCATTGATGAACTGA
GhARF6-F	GATCCTTGGCCGGAGTTTGT
GhARF6-R	GGCTCGCATAGTCGTCACAA
GhARF18-1-F	CGTACCAAGGTACATATGCAAGG
GhARF18-1-R	ACAACCGTCCCTTCACACTCC
GhARF18-5-F	GGAACAAACGAACACAACGGTTG
GhARF18-5-R	CTTGCAAAAATCCATCCATGG
GhYUC2-F	ACCGATGTGGGTTTTGGCGAAT
GhYUC2-R	CTCAGCATTTTCCCCGGTAGCA
GhYUC22-F	ACAACGCATCCCTTCGACACAA
GhYUC22-R	ACAGACCGACGCAGTACAAACC
GhCYP71A13-F	CGTAAACAGACCGAAACGCAGC
GhCYP71A13-R	GTGGTTGCGGAAAAGAGTTCGC
